# State of the Art of Genetic Engineering in Potato: From the First Report to Its Future Potential

**DOI:** 10.3389/fpls.2021.768233

**Published:** 2022-01-10

**Authors:** Vanesa Nahirñak, Natalia I. Almasia, Matías N. González, Gabriela A. Massa, Cecilia A. Décima Oneto, Sergio E. Feingold, Horacio E. Hopp, Cecilia Vazquez Rovere

**Affiliations:** ^1^Instituto de Agrobiotecnología y Biología Molecular, UEDD INTA-CONICET, Hurlingham, Argentina; ^2^Laboratorio de Agrobiotecnología, IPADS (INTA – CONICET), Balcarce, Argentina; ^3^Facultad de Ciencias Agrarias, Universidad Nacional de Mar del Plata, Balcarce, Argentina; ^4^Facultad de Ciencias Exactas y Naturales, Universidad de Buenos Aires, Buenos Aires, Argentina

**Keywords:** potato, genetic engineering, biotechnology, *Agrobacterium*, New Breeding Techniques, genome editing

## Abstract

Potato (*Solanum tuberosum* L.) is a crop of world importance that produces tubers of high nutritional quality. It is considered one of the promising crops to overcome the challenges of poverty and hunger worldwide. However, it is exposed to different biotic and abiotic stresses that can cause significant losses in production. Thus, potato is a candidate of special relevance for improvements through conventional breeding and biotechnology. Since conventional breeding is time-consuming and challenging, genetic engineering provides the opportunity to introduce/switch-off genes of interest without altering the allelic combination that characterize successful commercial cultivars or to induce targeted sequence modifications by New Breeding Techniques. There is a variety of methods for potato improvement *via* genetic transformation. Most of them incorporate genes of interest into the nuclear genome; nevertheless, the development of plastid transformation protocols broadened the available approaches for potato breeding. Although all methods have their advantages and disadvantages, *Agrobacterium*-mediated transformation is the most used approach. Alternative methods such as particle bombardment, protoplast transfection with polyethylene glycol and microinjection are also effective. Independently of the DNA delivery approach, critical steps for a successful transformation are a rapid and efficient regeneration protocol and a selection system. Several critical factors affect the transformation efficiency: vector type, insert size, *Agrobacterium* strain, explant type, composition of the subculture media, selective agent, among others. Moreover, transient or stable transformation, constitutive or inducible promoters, antibiotic/herbicide resistance or marker-free strategies can be considered. Although great efforts have been made to optimize all the parameters, potato transformation protocols are highly genotype-dependent. Genome editing technologies provide promising tools in genetic engineering allowing precise modification of targeted sequences. Interestingly, transient expression of genome editing components in potato protoplasts was reported to generate edited plants without the integration of any foreign DNA, which is a valuable aspect from both a scientific and a regulatory perspective. In this review, current challenges and opportunities concerning potato genetic engineering strategies developed to date are discussed. We describe their critical parameters and constrains, and the potential application of the available tools for functional analyses or biotechnological purposes. Public concerns and safety issues are also addressed.

## Importance of Biotechnology Application in Potato

Potato (*Solanum tuberosum* L.) is a worldwide important crop plant that produces high nutritional quality tubers. It is the fourth most important staple crop (after wheat, corn, and rice) in terms of production and demand, with around 378 million tons produced annually (Campos and Ortiz, 2020). The tuber, which serves the plant as a storage organ and as a vegetative propagation system, is an excellent source of complex carbohydrates, proteins, and vitamins (Banerjee et al., 2006; Barrell et al., 2013). Therefore, it is considered one of the promising crops to overcome the challenges of poverty and hunger worldwide (Bakhsh, 2020). In addition, potato is largely used in industry to make processed food products, alcohol, animal feed, and for bioenergy production substrates like biofuel (Ahmed et al., 2018). Moreover, given the physicochemical properties of refined starch it is used as a thickening and stabilizing agent in food, and as a raw material in paper, textile, cosmetic, adhesive, and plastics industries (Craze et al., 2018). Altogether, the importance of potato cultivation lies not only in its use as a basic food crop but also as a source of compounds of interest. While wide climate adaptability has facilitated potato to be extensively distributed in the world, several factors like climate change, industrialization, and urbanization have overburdened the existing agriculture lands and food resources (Badami and Ramankutty, 2015; Tiwari et al., 2020). Moreover, potato production faces important challenges such as biotic (viruses, bacteria, fungal, and insect pests) and abiotic stresses (drought, flooding, salinity, heat, and cold), and postharvest problems (accumulation of reducing sugars during cold storage, injury-induced enzymatic browning). Due to its large negative impacts on yield and tuber quality, improving resistance to disease and pests and/or abiotic factors, as well as quality traits is of significant economic importance (Rooke and Lindsey, 1998; Halterman et al., 2016).

Potato is a relevant candidate for improvement through conventional breeding and biotechnology. However, given that most of cultivated potatoes have tetraploid genomes, a high level of genetic heterozygosity and a narrow genetic base, conventional breeding is time-consuming and challenging (Chakravarty et al., 2007).

Therefore, genetic engineering provides the opportunity to introduce genes of interest without altering the allelic combinations that characterize successful commercial cultivars. Moreover, transgene-mediated post-transcriptional gene silencing using RNA interference (RNAi) is a strategy used to inactivate one or several genes and it has also proven its potential to obtain a desirable phenotype for potato improvement (Bradshaw, 2021). More recently, the so-called New Breeding Techniques (NBTs), which include genome editing, enabled the induction of targeted sequence modifications within a genome reducing off target effects.

Interestingly, several of potato genotypes are amenable to propagation through tissue culture enabling the application of available biotechnology techniques (Han et al., 2015; Halterman et al., 2016; Nadakuduti et al., 2018). Through genetic engineering approaches, different traits related to stress tolerance, nutritional quality, and/or compounds of interest were incorporated. Genetically engineered (GE) potato plants, obtained by classical genetic transformation strategies or genome editing tools, with increased resistance to insects, bacteria, fungi, viruses, herbicides, abiotic factors, and/or improved nutritional and post-harvest quality were developed. Also, the production of compounds such as biopharmaceuticals, biopolymers, polyhydroxyalkanoates, spiderweb, freeze-thaw-stable potato starch, increased synthesis of lipids and even vaccines, and human proteins were reported (for reviews see Pribylova et al., 2006; Halterman et al., 2016; Hameed et al., 2018; Rakosy-Tican and Molnar, 2021). Otherwise, the elimination of antinutritional or allergenic molecules such as alkaloids, glycoproteins, acrylamide, or patatin was also addressed (Zaheer and Akhtar, 2016; Hameed et al., 2018).

The large number of scientific reports and the diversity of engineered traits successfully transferred to potato reflect the ease of this crop to be biotechnologically improved. In this regard, the availability of potato genome sequence (Xu et al., 2011^1^) has facilitated the development of comparative genomic analyses and functional studies of candidate genes to improve several important traits.

In summary, genetic engineering methods are essential tools in plant science research, not only for applied but also for basic purposes, providing invaluable tools for the characterization and validation of gene function to better understand plant physiology and development. In this review, several biotechnological strategies applied to potato are discussed, from classic genetic transformation to genome editing.

## Strategies for Potato Transformation

Successful plant transformation requires a suitable DNA delivery system, an efficient plant regeneration protocol and an optimized selection method to recognize transgenic cells (Bakhsh, 2020). There is a wide variety of DNA delivery systems for the genetic transformation of potato. Most of them incorporate the genes of interest into the nuclear genome. However, in the recent years, the development of plastid transformation protocols has been reported, broadening the available approaches. A comprehensive view of the evidence reviewed, suggests that although all systems have their advantages and disadvantages, *Agrobacterium tumefaciens*-mediated transformation is the most used. Potato was one of the first crops to be successfully transformed with this method (Ooms et al., 1986) and since then, numerous genetic transformation protocols based on *A. tumefaciens* have been developed. However, effective alternative direct DNA uptake methods have been also reported (Halterman et al., 2016). The *A. tumefaciens* method is more efficient than others and results in a higher proportion of plants with a single transgene copy insertion, minimizing the potential side effects (Gelvin, 2017). Since the modification of traits based on multiple genes *via A. tumefaciens* is time-consuming and laborious (Romano et al., 2001), particle bombardment is the method of choice for transforming potato with several genes (Zhang et al., 2020). However, the stacking of transgenes *via A. tumefaciens* was successfully achieved through different approaches: crossing of individual transgenic plants, re-transformation with independent genetic constructs, use of gene combining constructs, co-transformation with double constructs, and the use of polyprotein systems, among others (Romano et al., 2001; Asurmendi, 2002; Rivero et al., 2012; Fernandez Bidondo et al., 2019). In addition, another biotechnological approach successfully applied in potatoes for stacking resistance genes against viruses is RNAi technology (Missiou et al., 2004; Chung et al., 2013; Hameed et al., 2017). It is important to note that RNAi approach has been successfully employed in a great number of reports for disease and pest resistance (Waterhouse et al., 1998; Missiou et al., 2004; Chung et al., 2013; Dinh et al., 2015; Sun et al., 2016; Hameed et al., 2017), improved processing quality (Rommens et al., 2006; Llorente et al., 2010; Chawla et al., 2012; Zhu et al., 2016; Hameed et al., 2018), and improved nutritional value (Van Eck et al., 2007; Itkin et al., 2013; Sawai et al., 2014). In fact, the effectiveness of this technology is illustrated in the several commercially approved GE potatoes based on RNAi (ISAAA, 2021^2^).

Generation of transgenic potato using other bacteria species, such as *Sinorhizobium meliloti*, *Rhizobium* sp. NGR234, *Mesorhizobium loti*, *Sinorhizobium adhaerens*, and *Agrobacterium rhizogenes*, has also been reported (Wendt et al., 2011, 2012; Butler et al., 2020). *A. rhizogenes* is often applied for obtaining transgenic roots faster than using *A. tumefaciens*, since plant regeneration is not needed, representing an alternative tool for potato functional studies and characterization of root genes (Fernández-Piñán et al., 2019).

Lastly, DNA delivery can be achieved by direct DNA uptake, like microinjection, particle bombardment, protoplast transfection with polyethylene glycol (PEG), and protoplasts electroporation (Fehér et al., 1991; Romano et al., 2001, 2003; Valkov et al., 2011). These methodologies would facilitate the coordinated integration and expression of several genes and the manipulation of metabolic pathways in potato (Craig et al., 2005). Even though these methods are effective (Weiland, 2003), disadvantages such as complex integration patterns, high copy numbers, transgene rearrangements, and gene silencing have also been reported (Sawahel, 2002). New methodologies for genetic engineering such as nanoparticle-mediated approaches for passive delivery of genetic cargo were successfully employed in potato (Abdel-Razik et al., 2017).

Once DNA was delivered, transient or stable transformation can occur. Virus induced gene silencing is an attractive approach to generate transient loss-of-function assays to assess the role of genes in a short time, as an alternative to stable transformation (Brigneti et al., 2004; Faivre-Rampant et al., 2004; Dobnik et al., 2016). In addition, *A. tumefaciens*-mediated infiltration without involving a viral based system has been reported to study gene function or protein localization in potato (Bhaskar et al., 2009). Although there are some reports using these tools in potato, their applications are far from routine and currently more limited to model plant species.

Other strategies involving DNA delivery to organelles have been employed. Plastid transformation has some potential advantages in comparison to nuclear transformation for both plant breeding and molecular farming. In contrast to the random nature of nuclear transformation, the gene of interest is integrated into the plastome through homologous recombination, which avoids negative effects associated with transgene insertion in transcriptionally silent regions or with the disruption of host genes or regulatory regions (Gelvin, 2017). Moreover, mechanisms of gene silencing are not present in plastids, so expression of the transgene is stable in progeny of transplastomic plants (Sidorov et al., 1999; Thanh et al., 2005). In addition, other advantages include high expression levels of transgenes and protein accumulation, the opportunity of expressing several genes in operons and the inherent confinement of transgenes and recombinant products in plastids (Valkov et al., 2011, 2014; Segretin et al., 2012). The availability of the complete chloroplast genome sequence of *S. tuberosum* in 2005, allowed the construction of species-specific vectors by increasing homology and the improvement of potato plastid transformation efficiency (Scotti et al., 2011; Valkov et al., 2011). Even though it has attractive advantages and potential applications in potato biotechnology, low transformation frequencies and the reduced levels of transgene expression registered in tubers limit a wider use of potato plastid transformation (Sidorov et al., 1999; Thanh et al., 2005; Segretin et al., 2012).

In sum, DNA delivery methods based on biological vectors –such as bacteria or virus–, physical agents –like electroporation, microinjection, or particle bombardment–, and chemical agents –such as PEG– are available for potato transformation. Even direct strategies are effective *A. tumefaciens*-mediated transformation is the most established and preferred approach for potato. The choice of the potato transformation strategy is based on the experimental objective, such as transient or stable integration and/or nuclear or plastid expression, and will determine the most suitable DNA delivery method.

## Main Parameters Influencing Transformation Efficiency

There is not a consensus in the definition of transformation efficiency in the bibliography, but we consider the most suitable estimation as follows: Transformation efficiency (%) = (Total number of PCR positive lines/Total number of inoculated explants) × 100. It is important to note that to calculate the exact efficiency only independent lines must be considered. According to the reviewed reports the most relevant parameters that affect potato transformation efficiency are detailed below.

### Potato Genotypes

There are more than 4,000 landraces of native potatoes, mostly found in the Andean Region and over 180 wild potato species (source: International Potato Center). Despite the great number of potato varieties available, few cultivars are commercialized, chosen for their viability to be marketed and stored (Yang et al., 2015). Cultivars ‘Desiree’ and ‘Bintje’ were early favorites for transformation assays but nowadays, most potato cultivars are amenable to tissue culture and nearly all commercially important varieties can be successfully transformed with modifications of the standard protocols (Vinterhalter, 2008; Han et al., 2015; Halterman et al., 2016; Nadakuduti et al., 2018; Bruce and Shoup Rupp, 2019; Kaur et al., 2020). In 1988, it was reported the development of a genotype-independent method to transform four potato cultivars (‘Bintje’, ‘Berolina’, ‘Desiree’, and ‘Russet Burbank’) using leaf discs (De Block, 1988). However, subsequent evidence showed that the transformation efficiency is dependent on genotypes. There are several reports comparing the regeneration and transformation efficiencies of different genotypes using the same protocol and the results indicate that the effectiveness in obtaining transgenic plants is variable (Conner et al., 1992; Dale and Hampson, 1995; Kumar et al., 1995; Heeres et al., 2002; Han et al., 2015; Bakhsh, 2020). Moreover, Heeres et al. (2002) determined that regeneration and transformation efficiency are two different genetically controlled factors. So, for a given genotype, the success of transformation depends on several critical factors including vector, *Agrobacterium* strain, infection time, mode of injury, pre-culture period, cocultivation time, explant type, composition of the subculture media, selection markers, among others (Kaur and Devi, 2019; Bakhsh, 2020). Thus, standard protocols can be considered to transform a new genotype but then all parameters should be optimized to achieve adequate efficiency.

### *Agrobacterium tumefaciens* Strains

Once the use of a bacterial delivery method has been defined, the next parameter to determine is the strain to use since it affects the efficiency of the genetic transformation. In this sense, LBA4404 *A. tumefaciens* strain is the most used in potato transformation protocols (Stiekema et al., 1988; Tavazza et al., 1989; Trujillo et al., 2001; Heeres et al., 2002; Bakhsh, 2020; Mollika et al., 2020). However, alternative strains such as EHA105, GV2260, GV3101, and C58C1 have also been successfully used (Kumar et al., 1995; Banerjee et al., 2006; Han et al., 2015; Ahmed et al., 2018; Craze et al., 2018; Bruce and Shoup Rupp, 2019; Décima Oneto et al., 2020). Despite the existence of numerous reports, there is not a clear association between the strain and the genotype to be transformed; this means that for a particular genotype the most suitable strain must be experimentally tested.

Once the strain was selected there are three principal factors that affect transformation efficiency: the optical density of the bacterial suspension, the addition of a centrifugation step of the bacterial culture before inoculation and the length of the co-cultivation period. Considering the evidence reviewed, it seems that the optimal optical density values are between 0.5 and 0.8, depending on the construct and the growth medium. Some protocols indicate that centrifugation of the bacterial culture before inoculation may affect the viability of the bacteria, causing a drastic reduction of transformation efficiency (Beaujean et al., 1998; Banerjee et al., 2006; Décima Oneto et al., 2020). Co-cultivation period should be long enough to enable proper T-DNA transfer, but prolonged periods should be avoided to reduce tissue damage and somaclonal variation. Therefore, the optimal time is between 24 and 96 h, being 48 h the most reported co-cultivation period (Millam, 2006). Nonetheless, for each genotype is necessary to determine empirically not only the optimal strain but also the co-cultivation conditions to obtain the best transformation efficiency.

### Vectors

Another important choice is the vector to employ based on the transformation strategy selected. The type, the size, the regulatory elements, the selectable marker gene, the cloning efficiency, the cost and the availability, among other factors, must be considered.

The type and the size of the vector are directly related to the transformation strategy selected, mainly whether a shuttle (see Hellens et al., 2000) or a not binary vector is necessary. Regarding the regulatory elements, the constitutive and ubiquitous CaMV35S promoter is commonly used to express transgenes in potato; however, there are many reports of promoters successfully employed for specific objectives (Yevtushenko et al., 2004; Pino et al., 2007; Li et al., 2015; Nahirñak et al., 2019). Moreover, regarding a cisgenic approach, well characterized tissue-specific or inducible promoters have been reported from potato (Martini et al., 1993; Naumkina et al., 2007; Almasia et al., 2010). Another point to consider in the vector design is to avoid repeated regulatory sequences to prevent possible silencing effects.

The selectable markers generally used to identify transformed plant cells are genes encoding resistance to antibiotics or herbicides (Bruce and Shoup Rupp, 2019). The neomycin phosphotransferase II (*nptII*) gene, which confers resistance to the antibiotic kanamycin, is the most used in potato transformation protocols (Barrell et al., 2013). Barrell et al. (2002) evaluated the efficiency of different selectable markers using identical vectors for potato transformation. The effectiveness of recovery of transgenic lines was ranked as follows: kanamycin resistance > hygromycin resistance > phosphinothricin resistance > phleomycin resistance > methotrexate resistance (Barrell et al., 2002).

Other markers have been reported for potato transformation that do not involve the use of herbicides or antibiotics and use xylose or galactose as selective agents instead (Haldrup et al., 1998; Joersbo et al., 2003). These systems offer alternatives to the conventional ones, although their use requires further optimization. Visual markers genes such as glucuronidase, luciferase and green fluorescent protein have also been adopted, although to a lesser extent (Sidorov et al., 1999; Verhees et al., 2002; Rakosy-Tican et al., 2007). Alternatively, marker-free transformation of potato has also been reported. This approach is based on the recovery of transformed plants by PCR screening of plants regenerated without the use of a selection system (De Vetten et al., 2003; Ahmad et al., 2008). However, this approach resulted in the recovery of transgenic potato lines at low frequencies, with most of lines displaying insertions of undesirable vector backbone sequences and only a few lines containing the desirable single T-DNA insertion, which is another important criterion for commercialization of GE crops (Kondrák et al., 2006).

Concerning *A. tumefaciens* binary vectors, the arrangement of expression cassettes in the plasmid should also be contemplated. The selectable/screenable markers should be near the left border to facilitate the selection of events containing the entire cassette since the transference of the T-DNA is directed from right border to left border. Also, the inwards opposite orientation of the cassettes should be avoided preventing possible silencing effects.

Finally, it is important to check the vector through DNA sequencing before starting the transformation protocol.

### Explants

For a genetic transformation system to be effective, it is essential to develop a rapid and efficient regeneration protocol. Leaves, stems, tubers, petiole, protoplasts, and micro-tubers have been used as explants to develop transgenic potato lines.

The source of explant tissue for potato transformation is frequently derived from *in vitro* plants (Barrell et al., 2013). The main advantage of using them is the supply of uniform and pathogen-free material for transformation (Newell et al., 1991). *In vitro* micropropagation of shoot cultures makes possible the accessibility of healthy and vigorously growing plant material throughout the year (Visser, 1991). Since it is sterile and already acclimatized to grow under *in vitro* conditions, surface sterilization of the plant tissue is not required, reducing both handling time and the possibility of contamination, and avoiding plant stress due to chemical treatments (Conner et al., 1992; Kumar, 1995).

Leaves and stem internodes from *in vitro* plants are the most widely used explants since they are readily available and easy to use. It has been reported that stem pieces are relatively robust and can be handled easily in larger numbers comparing to leaf explants, which are delicate and can be injured during the manipulation reducing the frequency of transformation and regeneration (De Block, 1988; Newell et al., 1991).

The main disadvantage of employing leaf and stem explants in transformation assays is the somaclonal variation which can occur in the callus phase (Visser, 1991). The use of tubers is advantageous because the possibility of somaclonal variation is reduced (Ishida et al., 1989). *In vitro* grown microtubers have several advantages over soil-grown tubers since they are derived from virus-free, aseptically grown potato shoots, they can be produced conveniently at any time in large quantities, and they take up less storage (Kumar, 1995).

It is important to note that a critical factor influencing the frequency of callus and regeneration is the physiological state of the starting material (Chakravarty and Wang-Pruski, 2010). To ensure a successful transformation, the selection of young and healthy explants from stock plant is preferred while damaged tissue will negatively affect regeneration potential (Ahmed et al., 2018; Craze et al., 2018). Moreover, the size of the explant should be large enough to resist *Agrobacterium* co-cultivation or biolistic bombardment without losing moisture during transformation procedures. Frequently the explant chosen for transformation depends on the cultivar or is determined by the experience of each laboratory (Barrell et al., 2013).

In conclusion, there are many options regarding tissue amenable for potato transformation, in any case it must be a healthy material, sterile, appropriate for manipulation and with regenerative capacity to obtain good and reproducible results.

### Tissue Culture Media

Most protocols employ a two-step regeneration procedure, with a callus induction step followed by a shoot growth step. In particular, the de-differentiation and redifferentiation of explants is one of the main points of an effective plant transformation (Zhang et al., 2020). There are many types of plant tissue culture media, most based on Murashige and Skoog’s MS Media (Murashige and Skoog, 1962). MS media contains the major salts, a variety of minor salts, sucrose, vitamins, and plant growth regulators, which include cytokinins, auxins, gibberellins, abscisic acid, and ethylene (for details about regulators used in potato tissue culture see Bruce and Shoup Rupp, 2019). The callus induction stage is often facilitated by treatment of explants with zeatin or zeatin riboside with low levels of auxin, while the shoot induction stage often has a reduction of zeatin and auxin, plus the addition of gibberellin to stimulate shoot outgrowth. Regeneration rates per explant are usually high and the first shoots appear after 4–6 weeks (Millam, 2006), depending on the genotype used. For each potato genotype, it is necessary to adjust and determine the ratio of growth regulators most suitable for dedifferentiation and form callus, which also depends on the explant used (Zhang et al., 2020). Furthermore, another factor that influences the different transformation steps is light types, intensity and photoperiod (Gelaye, 2014).

On the other hand, the success of genetic transformation relies not only on the DNA delivery approach and a rapid and effective regeneration protocol, but also on the selection system to identify the transgenic cells. An efficient selection system results from a delicate balance between the inhibition of growth of wild-type cells and the preservation of the regeneration capacity of transformed cells. It is important to determine empirically the lowest level of the selective agent that prevent the development of non-transformed cells under selective conditions to reduce the recovery of false positives (also named “escapes” since they have escaped the selection process). Moreover, if the selection pressure is too high it may result in false negatives because of the loss of transformed plants (Barrell et al., 2002). Even though several selection strategies have been reported for potato transformation, kanamycin resistance is the most used selectable system and was shown to be more effective in the rapid recovery of large numbers of independently derived transgenic lines (Barrell et al., 2002). It has also a history of safe use in 122 transgenic crops approved for cultivation, food, or feed (ISAAA, 2021).

The freshness of the media and growth regulators is relevant so they must be replaced regularly. Moreover, experiments controls are very important since they allow to identify problems, for example, non-inoculated pieces of leaves placed with a selection agent to demonstrate their functionality, and without selection to ensure that the shoots can regenerate. The experience acquired to identify the putative transgenics trough visual selection is another main point in potato transformation protocols, for example, the formation of roots in the selective medium is an excellent indicator of the existence of transformed tissues.

As it was already mentioned, one of the main constrain in potato genetic engineering, is the somaclonal variation, which consists of phenotypic changes observed when plants are regenerated from cultured somatic cells. The observed phenotypic variations among somaclonal potato lines involve physiological, epigenetic, and/or genetic changes (Barrell et al., 2013). Maintaining potato lines in culture for prolonged periods can result in these variations (Craze et al., 2018). Genotype, explant origin and the culture conditions are others critical variables contributing to somaclonal variation (Meiyalaghan et al., 2011). The success of gene transfer techniques also depends on minimizing these variations (Stiekema et al., 1988), especially by decreasing the callus induction stage (Millam, 2006) or the period of *in vitro* culture in general (Han et al., 2015).

As it is shown in Table 1 even though there are many potato transformation protocols reported for different genotypes most of them are based on *A. tumefaciens*, employ *nptII* as selective marker, and use leaves and stems as tissue explant.

**TABLE 1:** Examples of potato transformation protocols.

Method	Genotype	Explant	Selection marker	References
*Agrobacterium tumefaciens*	Two *S. tuberosum* cultivars; tetraploid line, cv. Russet Burbank, and diploid line	Leaf and stem	NPTII	An et al., 1986
	cv. Pentland Dell, cv. Desiree, cv. Maris Piper, cv. Maris Bard, and cv. Golden Wonder	Tuber disks	NPTII	Sheerman and Bevan, 1988
	cv. Bintje and cv. Desiree	Tuber disks	NPTII	Stiekema et al., 1988
	cv. Bintje, cv. Berolina, cv. Desiree, and cv. Russet Burbank	Leaf	NPTII	De Block, 1988
	cv. Desiree	Leaf	NPTII	Tavazza et al., 1989
	Diploid (6) and tetraploid (3) potato genotypes	Leaf and stem	NPTII	Visser et al., 1989
	cv. Russet Burbank and cv. Lemhi Russet	Microtuber discs	NPTII	Ishida et al., 1989
	cv. Russet Burbank	Stem	NPTII	Newell et al., 1991
	cv. Dam Hardy, cv. Iwa, and cv. Rua	Leaf	NPTII	Conner et al., 1992
	*Solanum verrucosum, Solanum hjertingii, Solanum papita, Solanum stoloniferum*, and *Solanum demissum*	Microtubers	NPTII and HPTII	Kumar et al., 1995
	cv. Desiree and cv. Pentland Squire	Leaf and stem	NPTII	Kumar, 1995
	cv. Desiree, cv. Bintje, and cv. Kaptah Vandel	Internodes	NPTII	Beaujean et al., 1998
	cv. Diacol, cv. Capiro, and cv. Parda Pastusa	Leaf	NPTII	Trujillo et al., 2001
	E-potato 3 and Guannongshu-2	Tuber disc	NPTII	Si et al., 2003
	*S. tuberosum* L. ssp. andigena line 7540	Leaf	NPTII	Banerjee et al., 2006
	cv. Shepody	Leaf and stem	NPTII	Gustafson et al., 2006
	cv. Desiree	Internodes	NPTII	Millam, 2006
	Dihaploid s 178/10, 224/1, and 227/5; cv. Desiree, cv. Agave, and cv. Delikat	Leaf and stem	NPTII/gfp	Rakosy-Tican et al., 2007
	cv. Bintje	Stem	NPTII	Chakravarty and Wang-Pruski, 2010
	cv. Cardinal and cv. Heera	Leaf and internodes	NPTII	Khatun et al., 2012
	cv. Jowon and cv. Atlantic	Leaf and stem	bar	Han et al., 2015
	cv. Innovator, cv. Marabel, var. Tokat-10/1 and var. Tokat-6/24	Leaf discs	NPTII	Ahmed et al., 2018
	cv. Desiree	Leaf pieces	NPTII	Craze et al., 2018
	cv. Desiree, cv. Ranger Russet, cv. Umatilla Russet, cv. Alturas, and cv. Yukon Gold	Stem	NPTII and HPTII	Bruce and Shoup Rupp, 2019
	cv. Kufri Chipsona	Leaf and internodes	NPTII	Kaur et al., 2020
	cv. Asterix	Internodes and microtuber discs	NPTII	Mollika et al., 2020
	cv. Lady Olympia, cv. Granola, cv. Agria, cv. Désirée, and cv. Innovator	Leaf discs and internodes	NPTII	Bakhsh, 2020
	cv. Spunta	Leaf	HPT	Décima Oneto et al., 2020
*Agrobacterium rhizogenes/A. tumefaciens*	cv. Désirée/cv. Désirée, ssp. andigena	Plants/leaf	NPTII	Fernández-Piñán et al., 2019
*A. rhizogenes*	cv. King Edward, cv. Record, Majestic, cv. Maris Bard, cv. Desiree, cv. Pentland Crown, and cv. Maris Piper	Stem	Not specified	Ooms et al., 1986
*Sinorhizobium meliloti, Rhizobium* sp. NGR234, and *Mesorhizobium loti*	cv. Maris Peer	Internodes	HPT	Wendt et al., 2011
Particle bombardment	cv. Désirée	Chloroplast (young leaves)	aadA	Valkov et al., 2014
Particle bombardment	*S. tuberosum*, genotype 1024-2	Internodes, leaves, and microtubers	NPTII	Romano et al., 2001
Particle bombardment/PEG	cv. Desiree	Leaf/protoplasts	HPT	Craig et al., 2005
Mg2+/PEG	cv. Gracia, cv. Desiree, and cv. Boro	Leaf protoplasts	NPTII	Fehér et al., 1991

## Genome Editing in Potato

In recent years, genome editing technologies have emerged as novel biotechnological approaches for crop breeding and have received considerable attention due to their simplicity and accuracy in introducing targeted modifications that result in desired traits (Arora and Narula, 2017; Baltes et al., 2017; Scheben et al., 2017; Gao, 2018; Chen et al., 2019; Zhu et al., 2020). Genome editing is based on the employment of site-specific nucleases (SSNs) to induce modifications at specific genomic sites (Gao, 2021). SSNs such as Zinc Finger Nucleases (ZFNs), Transcription Activator-Like Effector Nucleases (TALENs), and the more recently developed Clustered Regularly Interspaced Short Palindromic Repeats (CRISPR) and CRISPR associated proteins (CRISPR/Cas) introduce double stranded breaks (DSBs) at a specific target site in the host genome, which led to targeted modifications *via* endogenous DNA repair mechanisms (Schmidt et al., 2019). In somatic plant cells, DSBs are mainly repaired by the error-prone non-homologous end joining (NHEJ) pathway, which occasionally results in the introduction of small insertions or deletions at the repaired site, producing the disruption of specific genes and/or regulatory regions of the plant genome (Puchta, 2005). Although less frequent than NHEJ, the homology-directed repair (HDR) pathway can be triggered in plant cells to repair the induced DSBs (Schmidt et al., 2019). A requirement for the HDR pathway to take place is the availability of a homologous DNA fragment, which can be exploited for targeted integration of sequences of interest into the plant genome (Huang and Puchta, 2019).

The availability of potato genome sequence and the development of highly efficient transformation systems make potato a perfect candidate for the application of genome editing technologies to improve important traits leading to a more sustainable potato production (Hameed et al., 2018; Nadakuduti et al., 2018). Initial genome editing platforms, i.e., ZFN and TALEN, were developed through the fusion of a programmable DNA-binding domain (Zinc Fingers and Transcription Activator-Like Effectors for ZFN and TALEN, respectively) and the catalytic domain of the type II restriction enzyme *Fok*I (Voytas, 2013). Both platforms are based on protein-DNA interactions to recognize the target sequences, which represented a drawback in the widespread adoption of these technologies, due to the complexity in the design of the DNA-binding domains to recognize new sequences, their synthesis and activity validation (Isalan, 2012; Doudna and Charpentier, 2014). Nevertheless, TALEN technology has been successfully used for potato genome editing in several applications, including both basic potato research and the improvement of important traits (Table 2).

In contrast to ZFN and TALEN, the CRISPR/Cas systems utilize a short and programmable guide RNA molecule to recognize the target site, which represent a more simple, versatile and efficient platform to mediate genome editing (Chen et al., 2019). In particular, the components of the CRISPR/Cas9 system from *Streptococcus pyogenes* were the first to be adapted as a programmable genome-editing tool, consisting of a Cas9 nuclease directed by an easily re-programmable single guide RNA (sgRNA) (Jinek et al., 2012). Cas9 can induce DSBs at specific sites determined by both base complementary between the sgRNA and the target sequence and the presence of a 5′-NGG-3′ motif adjacent to the complementary region in the target sequence (PAM, protospacer adjacent motif) (Jiang and Doudna, 2017). The simplicity of CRISPR/Cas9 system made it the most widely applied technology for potato genome editing, as well as for the rest of plant species (Gao, 2021). Since the first report on a CRISPR/Cas9-mediated genome editing in potato in 2015 (Wang et al., 2015), this system has been applied in number of basic research studies and in improving important traits in potato, including its nutritional quality, modification of tuber starch composition, post-harvest quality enhancement, biotic stress tolerance, and elimination of reproductive self-incompatibility. Such applications have been extensively reviewed in recently published articles (Hameed et al., 2018; Nadakuduti et al., 2018; Dev et al., 2021) and are summarized in Table 2, together with more recently published reports.

### Genome Editing Reagents Delivery in Potato

Like in all plant species, delivery of the genome editing reagents in potato is based on previously established transformation methods (Ran et al., 2017). Therefore, *A. tumefaciens*-mediated transformation has been the most widely used approach to deliver either TALEN or CRISPR/Cas systems in potato (Table 2). Genomic integration of transgenes encoding the genome-editing reagents is effective in producing a sustained expression of the components that led to the intended modification of the target site(s).

**TABLE 2:** Applications of TALEN or CRISPR/Cas systems for both basic research and agronomic/agroindustrial traits improvement in potato.

Genome editing technology	DNA repair pathway	Delivery approach	Genotype	Target gene	Objective	References
TALEN	NHEJ	*Agrobacterium tumefaciens*	cv. Sassy	Sterol side chain reductase 2 (*SSR2*)	Functional genomics	Sawai et al., 2014
	NHEJ	Protoplast transfection with DNA vector	cv. Desiree	Acetolactate synthase (*ALS*)	Proof of concept	Nicolia et al., 2015
	NHEJ	Protoplast transfection with DNA vector	cv. Ranger Russet	Vacuolar invertase (*VInv*)	Nutritional quality. Reduction of cold-induced sweetening (CIS)	Clasen et al., 2016
	HDR/NHEJ	*Agrobacterium tumefaciens* transformation plus donor vector (m*StALS*)	cv. Ranger Russet	Acetolactate synthase (*StALS*)	Herbicide resistance. Targeted T-DNA integration.	Forsyth et al., 2016
	NHEJ	*Agrobacterium tumefaciens*	cv. Sayaka	Granule-bound starch synthase (*GBSS*)	Construction of a Gateway-assisted TALEN system	Kusano et al., 2016
	NHEJ	*Agrobacterium tumefaciens*-mediated agroinfiltration	cv. Shepody and cv. Russet Burbank	Granule-bound starch synthase (*GBSS*) and vacuolar invertase (*VInv*)	Proof of concept	Ma et al., 2017
TALEN and CRISPR/Cas9	NHEJ	*Agrobacterium tumefaciens* with either a conventional T-DNA or a modified geminivirus T-DNA	cv. Desiree and diploid self-incompatible breeding line, MSX914-10 (X914-10)	Acetolactate synthase (*ALS*)	Herbicide resistance	Butler et al., 2016
CRISPR/Cas9	NHEJ	*Agrobacterium tumefaciens*	DM	Phytoene desaturase (*PDS*) and *StAA2* gene (encoding an Aux/IAA protein)	Proof of concept	Wang et al., 2015
	NHEJ	*Agrobacterium tumefaciens* with either a conventional T-DNA or a modified geminivirus T-DNA	cv. Desiree and diploid self-incompatible breeding line, MSX914-10 (X914-10)	Acetolactate synthase (*ALS*)	Proof of concept	Butler et al., 2015
	NHEJ	*Agrobacterium tumefaciens*	cv. Desiree	Transcription factor gene *MYB44*	Functional genomics	Zhou et al., 2017
CRISPR/Cas9	NHEJ	Protoplast transfection with DNA vector	cv. Kuras	Granule-bound starch synthase (*GBSS*)	Modification of starch composition. High amylopectin.	Andersson et al., 2017
	NHEJ	*Agrobacterium tumefaciens*	*S. tuberosum* group Phureja S15-65 clone	S-locus RNase (*S-RNase*)	Elimination of reproductive self-incompatibility	Ye et al., 2018
	NHEJ	*Agrobacterium tumefaciens*	DRH-195 and DRH-310	S-locus RNase (*S-RNase*)	Elimination of reproductive self-incompatibility	Enciso-Rodriguez et al., 2019
	NHEJ	*Agrobacterium rhizogenes*	cv. Mayqueen	Steroid 16α-hydroxylase (*St16DOX*)	Nutritional quality. Reduction of toxic steroidal glycoalkaloids (SGAs)	Nakayasu et al., 2018
	NHEJ	Protoplast transfection with RNP	cv. Kuras	Granule-bound starch synthase (*GBSS*)	Modification of starch composition. High amylopectin.	Andersson et al., 2018
	NHEJ	*Agrobacterium tumefaciens*	cv. Sayaka	Granule-bound starch synthase (*GBSS*)	Optimization of Cas9 expression with d-Mac3 translational enhancer	Kusano et al., 2018
	NHEJ	Protoplast transfection with vector using PEG and *Agrobacterium tumefaciens*	cv. Desiree	Starch branching enzymes (*SBE1* and *SBE2*)	Modification of starch composition/nutritional quality. High amylose and longer amylopectin chains.	Tuncel et al., 2019
	NHEJ	Protoplast transfection with DNA vector	cv. Desiree and cv. Wotan	Granule-bound starch synthase (*GBSS*)	Modification of starch composition. High amylopectin.	Johansen et al., 2019
	NHEJ	*Agrobacterium tumefaciens*-mediated transient expression	cv. Desiree	Phytoene desaturase (*PDS*)	Proof of concept	Bánfalvi et al., 2020
	NHEJ	Protoplast transfection with RNP	cv. Desiree	Polyphenol oxidase 2 (*StPPO2*)	Post-harvest quality. Reduction of enzymatic browning.	González et al., 2020
	NHEJ	*Agrobacterium tumefaciens* and protoplast transfection with DNA vector and RNP	cv. Desiree	Polyphenol oxidase 2 (*StPPO2*)	Comparison of CRISPR/Cas9 delivery approaches	González et al., 2021
CRISPR/Cas9	NHEJ	*Agrobacterium tumefaciens*	cv. Desiree and cv. King Edward	S-genes (*StMLO1*, *StHDS*, *StTTM2*, *StDND1*, *StCHL1*, *StDMR6-1* and *StDMR6-2*)	Biotic stress tolerance. Resistance to *Phytophthora infestans*	Kieu et al., 2021
	NHEJ	Protoplast transfection with RNP	cv. Desiree	Starch branching enzymes (*SBE1* and *SBE2*)	Modification of starch composition/nutritional quality. High amylose and longer amylopectin chains.	Zhao et al., 2021
Cytosine base editor A3A-PBE: nCas9 fused to the APOBEC3A cytidine deaminase	Base editing	Protoplast transfection with DNA vector	cv. Desiree	Granule-bound starch synthase (*GBSS*) and acetolactate synthase (*ALS*)	Construction of A3A-PBE cytosine base editor	Zong et al., 2018
CRISPR/Cas9 and cytosine base editor Target-AID: nCas9 fused to the PmCDA1 cytidine deaminase	NHEJ and base editing	Protoplast transfection with DNA vector and *Agrobacterium tumefaciens*	cv. Desiree and cv. Furia	Granule-bound starch synthase (*GBSS*)	Modification of starch composition. High amylopectin.	Veillet et al., 2019a
Cytosine base editor Target-AID: nCas9 fused to the PmCDA1 cytidine deaminase	Base editing	*Agrobacterium tumefaciens*-mediated transient expression	cv. Desiree	Acetolactate synthase (*ALS*)	Herbicide resistance	Veillet et al., 2019b
*Staphylococcus aureus* CRISPR/Cas9 and cytosine base editor SanCas9: *Staphylococcus aureus* nCas9 fused to the PmCDA1 cytidine deaminase	NHEJ and base editing	*Agrobacterium tumefaciens*	cv. Desiree	Granule-bound starch synthase (*GBSS*) and Downy Mildew Resistant 6 (*StDMR6-1*)	Proof of concept	Veillet et al., 2020

*Unless otherwise indicated, CRISPR/Cas9 refers to the system derived from Streptococcus pyogenes.*

Transient expression of the genome editing reagents is a promising alternative delivery method to obtain transgene-free edited plants (Chen et al., 2019). This is of a special importance in a vegetatively propagated and highly heterozygous crop like potato since the elimination of the transgenes by crossing or self-crossing is not suitable. Transient expression of genome editing reagents has been achieved in potato using different strategies. Bánfalvi et al. (2020) have obtained edited potato plants of the tetraploid cultivar Desiree trough transient expression of a CRISPR/Cas9-coding vector upon *A. tumefaciens*-mediated infection by using a selection protocol consisting in 3 days-kanamycin treatments. Edited lines (2–10%) were screened to check the binary vector incorporation in their genomes by PCR amplification of specific vector fragments, with negative results in all analyzed cases (Bánfalvi et al., 2020). Nicolia et al. (2015) had developed a pipeline based on the transient expression of TALEN in potato by protoplast transfection with DNA vector followed by whole plant regeneration without selective agents, obtaining 10% of the regenerated lines with mutations in the expected region (Nicolia et al., 2015). In addition to the transient expression of DNA vectors, protoplasts allow the delivery of CRISPR/Cas9 components as pre-assembled ribonucleoprotein (RNP) complexes (Woo et al., 2015). This represents a promising alternative, with the potential of both reducing to a minimum (or even eliminating) the possibility of foreign DNA insertions in the plant genome and minimizing the probabilities of unwanted off-target effects. Such an alternative may result of great interest in the application of this technology to obtain potato commercial cultivars, since the obtained edited plants could be indistinguishable from those containing naturally or conventionally induced mutations (Kumlehn et al., 2018). The first application of RNP complexes to obtain potato edited lines was reported by Andersson et al. (2018). Authors transfected protoplasts isolated from the cultivar Kuras, with two different types of RNP complexes targeting the GBSSI gene and obtained a frequency of 1–25% of regenerated edited lines, varying with the transfection conditions and the origin of RNP complexes (Andersson et al., 2018). Furthermore, for one transfection up to 3% of the total analyzed lines displayed mutations in all four alleles of the target gene and no DNA insertions. Since this first report, RNPs have been successfully used to mediate potato genome editing using the protoplasts transfection and regeneration system, with editing efficiencies ranging from 27 to 68% (González et al., 2020) and 52 to 72% (Zhao et al., 2021), varying with the targeted genes and the design of RNP components. Protoplasts provide a suitable platform for genome-editing reagents delivery and may in some cases represent even a more efficient strategy when compared to *Agrobacterium*-mediated transformation (González et al., 2021). Even though it represents a promising strategy to mediate transgene-free genome editing in potato, the use of RNP in protoplasts present some important aspects to consider. For instance, DNA traces remaining in the assembled RNPs may led to unintended foreign DNA insertions in the regenerated plants, with different frequencies (Andersson et al., 2018; González et al., 2021; Zhao et al., 2021). A possible origin of such traces is the DNA molecules employed for the *in vitro* transcription of sgRNAs, before RNP assembly. To solve this issue, the use of synthetically produced sgRNAs to obtain the RNP complexes completely eliminates the foreign DNA insertions (Andersson et al., 2018). On the other hand, regeneration of complete potato plants from protoplasts has been associated with somaclonal variation and chromosome instability (Barrell et al., 2013; Fossi et al., 2019). Therefore, the selection of strategies that enhance the number of edited lines after protoplasts regeneration, ensures a vast number of individuals to select the best candidates for further phenotypic analysis (Andersson et al., 2017).

### New Clustered Regularly Interspaced Short Palindromic Repeats-Related Tools for Potato Genome Editing

Clustered Regularly Interspaced Short Palindromic Repeats/Cas systems have been rapidly expanding and new CRISPR-related tools have been created that greatly broaden the scopes of genome editing and/or made it more precise.

Base editing technology is based on the fusion of a catalytically inactive (or partially inactive) Cas nuclease, fused to a cytosine or adenosine deaminase domain capable to convert one base to another (Mishra et al., 2020). Cytosine base editors (CBEs) convert a C to a G, while adenosine base editors (ABEs) mediate the conversion of A to G. CBEs have been successfully used to mediate potato base-editing (Table 2). Moreover, CBE consisting in a Cas9 nickase (nCas9) fused to either a human (Zong et al., 2018) or a *Petromyzon marinus* cytidine deaminase (Veillet et al., 2019a,b) were reported to obtain loss-of-functions or gain-of-function mutations related to important traits in potato.

The use of CRISPR/Cas systems originated from other bacteria species than *S. pyogenes* provide new benefits, including but not limited to, the expansion of the putative target sites within a given genome *via* different PAM requirements and the generation of different cutting patterns to introduce the DSB (Huang and Puchta, 2021). In a proof-of-concept publication, Veillet et al. (2020) demonstrated that Cas9 nuclease derived from *Staphylococcus aureus* (SaCas9) is efficient in inducing both genome editing *via* the NHEJ repair pathway and precise base editing in potato. Since SaCas9 recognize a more complex PAM (5′-NNGRRT-3′) it could additionally represent a highly specific nuclease by limiting the off-target activity, particularly for highly conserved genomic sequences in polyploidy species, such as potato (Veillet et al., 2020). This and other CRISPR/Cas orthologs systems are available for potato genome editing and can expand the toolbox for trait improvement (Huang and Puchta, 2021).

The recently developed prime editing technology allows the creation of different types of genomic changes with high precision, which represents potentially a new technological breakthrough (Gao, 2021). Prime editors are composed of an nCas9 fused to a reverse transcriptase, guided to the target site *via* a modified prime-editing guide RNA. In addition to define the target site, the prime-editing guide RNA serves as a template for reverse transcription, carrying a primer binding site and a sequence to be copied in the genome at its 3′end. Once nCas9 nicks the DNA, the released ssDNA can hybridize with the primer binding site and be used as a primer for the reverse transcriptase, which transfers the sequence encoded in the prime-editing guide RNA to the DNA strand (Huang and Puchta, 2021). The new sequence is incorporated later into the target site, through the DNA repair. Although prime editing is still inefficient in plant cells and no reports with this technology has been published in potato so far, its applications in a few crop species and its continuous optimization rise high hopes to incorporate it to the potato genome editing toolbox (Lin et al., 2021).

### Public Concerns About Genetically Engineered Potato

As we already stated in this review, genetic engineering includes genetic transformation and genome editing. Genetic transformation comprises the traditional tools for introduction of a gene of interest randomly integrated into plant genomes. Genome editing techniques have been developed as an alternative to introduce precise and predictable genome modifications into plants without adding foreign DNA. For a GE potato to become a market success it must be accepted by government regulatory system, producers, and consumers (Halterman et al., 2016).

Nowadays, most countries practicing commercial agriculture have established regulations, with different degrees of stringency, for field experimentation and later larger scale cultivation of GE crops. These regulations take into consideration food, feed, and environmental safety risks. Some countries have a process-oriented regulation and have established that the regulations that apply to genetically modified organism should be also applied to genome editing developments (European Union, New Zealand) (Nadakuduti et al., 2018). Other countries have pronounced in favor of regulating the varieties obtained by genome editing as conventional ones, if the developed varieties lack from any foreign genetic material (Argentina, United States, Brazil, etc.) (Feingold et al., 2018; Lema, 2019).

After more than 20 years of biosafety analysis of different GE potatoes, the main concern still regards the potential genetic flow between cultivated and wild relatives plants, particularly in the centers of origin. In most of the major potato producing countries, the chance of gene flow to interfertile species is virtually non-existent due to the lack of wild relatives. In South America, the Andean region is the center of origin of potato and represents a valuable source of diversity, being an important genetic resource. Even though intercrossings between cultivated potato and wild relatives have been reported, it is commonly restricted by reproductive barriers (Hawkes and Hjerting, 1969; Rabinowitz et al., 1990). Thus, the risk associated with a commercial released of GE potato in regions where interfertile species coexist should be evaluated case by case. In Argentina, an interspecific out-crossing trial between a *S. tuberosum* cv. Spunta transgenic line and the only wild relative species in the Pampean region that could possibly cross with it (*Solanum chacoense*), failed to detect any event of transgene transfer under natural field conditions (Bravo-Almonacid et al., 2012). In addition to safety, public concern also focuses on patents, plant breeders’ rights and the concentration of intellectual property in a small number of corporations (Haverkort et al., 2016). These are some points, together with the economic cost of risk assessment and regulatory framework process, that the political leadership should include in their schedule to facilitate the social access of these technologies to their farmers.

Regarding the producers, they are who probably see, more directly, the great benefits of the use of GE potatoes in their production. Recently, Bangladesh started a field trial of two late blight resistant transgenic potato lines, the success of which will allow 20% less yield loss and farmers will save around $ 12 million spent on fungicides (source PotatoPro^3^). In addition, the avoidance of agrochemical uses and its impact on carbon balance that may affect climate change will lead to important environmental benefits. Also, in Uganda, another transgenic variety that resists late blight infection without the use of fungicides is being evaluated. This would improve the safety of farmers and their families, and in particular the small farmers, who have limited access to fungicides, could reduce their production losses by up to 60% (Potato News Today, 2021). According to Ghislain et al. (2021) delaying adoption of this potato will lead to continued pesticide use and significant losses that will affect the most vulnerable farmers. On the other hand, the improvement of GE plant resistance to abiotic stresses such as salinity, drought, or temperature allows potato cultivation in less fertile agricultural lands (Hameed et al., 2018). Nevertheless, looking closely at the history of approved GE potato it can be noticed that producers may be reluctant to incorporate improved GE varieties because the industry fears mixing them with conventional ones and thus losing markets (Grafius and Douches, 2008; Haverkort et al., 2016; Tagliabue, 2018). This industry decision responds to a pre-judgment of consumers reactions, or to avoid negative publicity of their products. However, a science based potential risks and benefits communication campaign should be undertaken to comply with consumers’ right to be informed.

Consumer apprehension toward GE technology exists even though the cultivation area of these crops in 2020 was around 190 million hectares all over the world. According to Mullins et al. (2006), consumer concerns toward these technologies focuses on the public’s desire for unbiased information about potential risks. This leads us to think about the need to communicate clear, precise, and comprehensible information about GE technologies to the public, particularly showing the great advantages on its use. Public debate on the release of GE crops has led to questions regarding their environmental safety; however, it is important to clearly transmit that the deregulation of GE crops involves risk assessments and a complex regulatory framework. Concerning ecological compatibility of GE potato, many studies focused to address biosafety aspects and risk assessment did not find undesired ecological side-effects (Griffiths et al., 2000; Heuer et al., 2002; Rasche et al., 2006; Bravo-Almonacid et al., 2012; Fernandez Bidondo et al., 2019; van der Voet et al., 2019). In addition, as we mentioned before, GE potatoes resistant to biotic factors allow a radical reduction of pesticide applications, which are harmful to the environment and human health. Other advantage of GE potato directly relevant for consumers is the elimination of anti-nutritional or allergens to improve the nutrition quality. Recently, the potato variety Z6, engineered for low reducing sugars, low acrylamide potential, reduced black spot bruising and late blight protection, was deregulated in United States (source: USDA, 2021). It is interesting to discover if this GE potato with direct nutritional benefits will be well received by consumers. According to the international database, there are currently 51 events recorded on the potato list (ISAAA, 2021) and hundreds in the regulatory or pre-commercialization pipeline. The public concerns of consumers must be allayed with the information necessary to understand that GE potato are important for the sustainability of potato production, food security, income generation, and environmental protection. Therefore, it is worth taking a proactive approach to consumer apprehension in which all actors involved in the generation and adoption of these technologies provide that accurate information.

**FIGURE 1:**
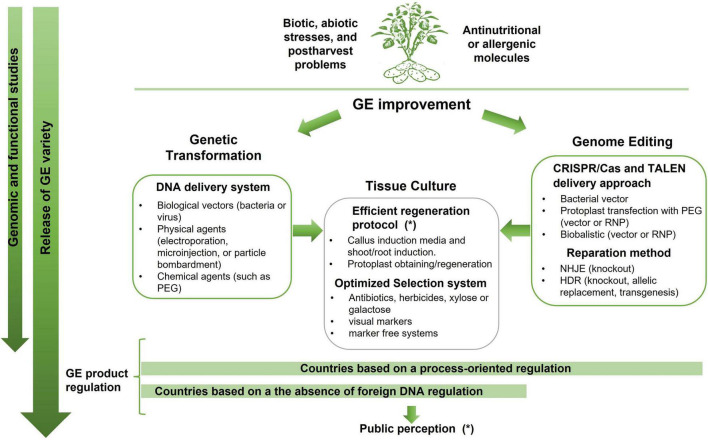
Simplified diagram of the strategies for genetic engineering improvements: genetic transformation or genome editing and common steps of vegetal culture to achieve it. Genetic transformation includes the traditional tools for introduction of a gene of interest randomly integrated into plant genomes. Genome editing techniques have been developed as an alternative to introduce precise and predictable genome modifications into plants to obtain desired traits. Those technologies were refinements of transformation whose final purpose is the obtaining a modified plant without foreign DNA. It is important to note that the regulations governing these developments vary from country to country. Some countries have a process-oriented regulation and apply the same regulation for all the GE products. Others consider the presence of foreign DNA as a mandatory requirement to be regulated and only in that case the product should be subjected to government regulations. Despite the differences between the classical genetic transformation strategies and the genome editing tools, both relies on tissue culture to regenerate and select GE plants. The symbol (*) represent the main constrains: the regeneration protocol for recalcitrant genotypes and the public perception regarding the release of GE varieties.

The Figure 1 summarizes the different strategies available for potato genetic engineering, including both genetic transformation and genome editing focusing on the common steps and the differences between them.

## Future Perspectives and Concluding Remarks

Potato is relatively easy to improve by genetic modification and genome editing because it benefits from a good amenability to regenerate shoots from *in vitro* tissue and protoplast culture. However, difficulties due to recalcitrance to transformation for some genotypes remain given that the limited regeneration capability *in vitro* restricts the recovery of transformed lines. The regeneration step is often the biggest bottleneck in the transformation process and is one of the most important factors explaining the low success in obtaining GE plants from recalcitrant genotypes. The entire plant regeneration from transformed explants involves tissue-culture procedures that can be time-consuming and can result in undesirable somaclonal variation.

The development and application of new technologies to overcome these drawbacks, especially bypassing tissue culture methods, are invaluable. In the recent years, some strategies to improve the regeneration efficiency in several plant species have been reported. These approaches involve the overexpression of genes encoding developmental regulators that can potentially be applied to improve plant transformation technologies (Debernardi et al., 2020; Maher et al., 2020; Kong et al., 2020). Interestingly, Maher et al. (2020) showed that the ectopic expression of specific transcription factors in somatic cells has the potential to induce meristems avoiding the use of traditional tissue culture. They demonstrated the induction of *de novo* meristems and the consequently shoot regeneration in various plant species including potato. Debernardi et al. (2020) reported that the expression of one Growth-Regulating Factor (GRF4) and its cofactor (GIF1) substantially increases regeneration efficiency in both monocotyledonous and dicotyledonous species; they even developed a protocol inducing embryogenesis in the absence of cytokinins. Finally, Kong et al. (2020) showed that the overexpression of GRF5 enhances regeneration and genetic transformation in various crop species. These advances represent a promising tool to overcome the problems associated with low regeneration capacities of certain genotypes improving the transformation process. However, the constitutive expression of developmental regulators not only enhances transformation efficiency but can also result in abnormal growth, so it is necessary to restrict/eliminate their expression in the plant after transformation. Even though these strategies would facilitate plant transformation in a broad range of recalcitrant genotypes new tools are needed such as the use of suitable promoters to control tissue- and timing-specific expression of these developmental regulators. In vegetatively propagated and highly heterozygous crops like potato, the procedure for developmental transgenes removal in subsequent generations through segregation is a challenge. We speculate that this last could be an option for some recalcitrant wild potatoes since most of them are diploid, so apart from asexual reproduction (by stolons and tubers), they have the alternative modes of sexual reproduction (by seeds) hence diploid progenies segregate for genes. In sum, the use of new technologies, would overcome the problems and limitations related to the classical methodology of potato transformation for some recalcitrant genotype.

There are many reports concerning successfully gene transfer of economically important genes in potato and GE varieties have been developed for a wide range of traits (Pribylova et al., 2006; Halterman et al., 2016; Zaheer and Akhtar, 2016; Hameed et al., 2018; Nadakuduti et al., 2018; Dev et al., 2021; Rakosy-Tican and Molnar, 2021). However, the use of biotechnology for potato improvement has been ultimately constrained by the regulation process and the public perception (Ghislain and Douches, 2020). Unfortunately, the consumer apprehension affects the field cultivation of the GE varieties and their adoption in the food chain. Looking closely at the history of approved potato GE, there are examples of deregulated potatoes whose field cultivation was discontinued due to lack of acceptance (Rakosy-Tican and Molnar, 2021; Bradshaw, 2021).

Along all the process from the development to the release of GE varieties, plant transformation and regeneration are the limiting factors for several crops, nonetheless for potato this does not seem to be the main constrain. The public perception is possibly the most difficult issue regarding the production and marketing a GE potato. The use of GE potato would allow reducing the use of pesticides, increasing yields, reducing production costs, lowering undesirable characters and/or providing a better nutrition quality, which would guarantee an adequate intake of vitally important foods. Taking everything into consideration and pondering the demographic expansion that is coming, it would be desirable that technical, ethical, and social/public constraints should be overcome in a relatively short time.

## Author Contributions

VN, NA, CV provided the outlines of the review and contributed the key ideas. VN, NA, MG, and GM wrote the manuscript and prepared the tables. CV, HH, GM, CD, and SF worked on and improved the original draft. All co-authors approved the manuscript.

## Conflict of Interest

The authors declare that the research was conducted in the absence of any commercial or financial relationships that could be construed as a potential conflict of interest.

## Publisher’s Note

All claims expressed in this article are solely those of the authors and do not necessarily represent those of their affiliated organizations, or those of the publisher, the editors and the reviewers. Any product that may be evaluated in this article, or claim that may be made by its manufacturer, is not guaranteed or endorsed by the publisher.

## Footnotes

1
http://spuddb.uga.edu/pgsc_download.shtml

2
https://www.isaaa.org/gmapprovaldatabase/default.asp

3
https://www.potatopro.com/es

## Abbreviations

ABEs: adenosine base editors
bar: herbicide bialaphos
CBEs: cytosine base editors
CRISPR: Clustered Regularly Interspaced Short Palindromic Repeats
DSBs: double stranded breaks
GE: genetically engineered
HDR: homology-directed repair
HPT: hygromycin phosphotransferase
NHEJ: non-homologous end joining
*nptII*: neomycin phosphotransferase II
PAM: protospacer adjacent motif
PEG: polyethylene glycol
RNP: assembled ribonucleoprotein
SSNs: site-specific nucleases
ZFNs: Zinc Finger Nucleases.
